# A novel electrochemical sensor based on MIP technology for sensitive determination of cinacalcet hydrochloride in tablet dosage form and serum samples

**DOI:** 10.1007/s00604-025-07152-7

**Published:** 2025-04-15

**Authors:** Ipek Kucuk, Selenay Sadak, Selda Zengin Kurnalı, Sacide Altınöz, Bengi Uslu

**Affiliations:** 1 Department of Analytical Chemistry, Faculty of Pharmacy, Başkent University, 06790 Ankara, Türkiye; 2 The Graduate School of Health Sciences, Ankara University, 06110 Ankara, Türkiye; 3 NOBEL Holding A.S. R&D Center, 81100 Duzce, Türkiye; 4 Department of Analytical Chemistry, Faculty of Pharmacy, Ankara University, 06560 Ankara, Türkiye

**Keywords:** Cinacalcet hydrochloride, Imprinting factor, Molecularly imprinted polymers, Pharmaceutical dosage form, Electrochemical sensor, Modified glassy carbon electrode, Differential pulse voltammetry

## Abstract

Cinacalcet hydrochloride (CIN) is a calcium-sensing receptor agonist used to treat hypercalcemia in the parathyroid. The molecularly imprinted polymer (MIP)–based sensor (CIN@MIP/GCE) was electropolymerized using cyclic voltammetry (CV) of the functional monomer o-phenylenediamine (o–PD) with a template molecule CIN on a glassy carbon electrode (GCE). The optimum performance of the MIP-based electrode for CIN detection was obtained with parameters of a 1:7 monomer ratio, a 15-min removal time, ethanol as a removal solution, and a 15-min rebinding time. The surface characterization of the CIN@MIP/GCE sensor was conducted using atomic force microscopy (AFM) and scanning electron microscopy (SEM), while CV and electrochemical impedance spectroscopy (EIS) were employed for electrochemical characterization with [Fe(CN)_6_]^3−^/^4−^ redox probe. AFM findings show that the MIP sensor with CIN-specific voids on the surface has a root-mean-square (RMS) value of 27.95, while the non-imprinted polymer (NIP) sensor without voids has a smoother surface formation and an RMS value of 21.30 nm. The analytical efficacy of the constructed sensor was assessed using differential pulse voltammetry (DPV). The limit of detection (LOD) was 0.17 × 10^−12^, with a linear range of 1.0 × 10^−12^–1.0 × 10^−11^ M. The reliability of the constructed sensor was determined using CIN detection in real samples as tablet dosage form and human serum, yielding recovery results of 100.19% and 101.82%, respectively. The selectivity investigation was performed against prevalent interference substances. The relative imprinting factor (IF) values of CIN impurities confirmed the selectivity of the CIN sensor.

**Graphical Abstract:**

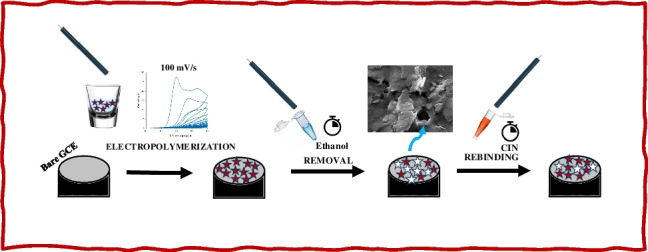

**Supplementary Information:**

The online version contains supplementary material available at 10.1007/s00604-025-07152-7.

## Introduction

CIN is a calcimimetic indicated for the management of secondary hyperparathyroidism in individuals with chronic kidney disease (CKD) undertaking dialysis, as well as for the treatment of hypercalcemia in patients with parathyroid cancer [1]. It helps prevent osteoporosis and cardiovascular complications by regulating parathyroid hormone (PTH) levels. It has high bioavailability and is metabolized by CYP3 A4 and CYP1 A2 enzymes. Its use as an oral formulation increases patient compliance and facilitates treatment processes. A decrease in serum Ca levels has been observed with the use of CIN, but a significant decrease may cause hypocalcemia in patients, which may lead to muscle cramps, seizures, and cardiac problems. Since the plasma half-life is 30–40 h, the drug has the potential to remain in the body for a long time. Detection at nanomolar levels is of significant importance to understand whether the level of the drug in plasma is within safe ranges. At the same time, the detection of CIN at nanomolar levels is a great necessity in terms of ensuring treatment effectiveness, preventing toxicity risks, monitoring drug interactions, monitoring drug accumulation in kidney/liver patients, and ensuring accuracy in bioequivalence studies.

Various methods such as liquid chromatography-mass spectrometry (LC–MS/MS) [2–5], reverse phase high-performance liquid chromatography (RP-HPLC) [6–9] UV via derivatization [10], and ultra-fast liquid chromatography (UFLC) [11] have been developed for the detection of CIN (Table S1). In 2020, Kumar et al. performed sensitive determination of CIN by reverse phase high-performance liquid chromatography. They obtained a linear range of 5–50 µg/mL and a lower LOD of 0.32 µg/mL and performed a recovery study in tablet dosage forms [6]. Apart from this study, there is only one electrochemical analysis study for the determination of CIN in the literature. In the other study, the interaction of CIN with DNA was discussed. The interaction studies indicated that the binding mode for the interaction may be groove binding, and the detection limit was calculated as 0.15 μg/mL[12]. Our study is not only selective compared to other studies but also provides a sensitive method with very low detection limits.

Electrochemical techniques are among the most widely used approaches due to their cost-effectiveness, reliability, practicality, and capacity to achieve much lower detection limits. At the same time, electroanalytical analyses have gained a wide linear range, good stability, and repeatability with the improved sensors. The most commonly used working electrode in electrochemical techniques is the GCE. Carbon-based electrodes are preferred over metal electrodes because of their lower cost and wider operating potential range [13]. Among carbon electrodes, GCE has smaller pores on its surface than carbon paste electrode and this provides more reproducible results. Compared to sonogel electrodes obtained by combining silica-based polymers and organic solvents, GCE is much more chemically resistant; in other words, it shows high stability [14, 15].

Electrodes are modified with various nanomaterials to obtain more precise measurements, more reliable results, and wider application areas in electrochemical analysis. In this study, the MIP technology with the highest selectivity and sensitivity compared to other materials was chosen as the modification. The most important advantage of MIPs is based on the creation of analyte-specific selective cavities on the polymer immobilized on the electrode surface and the rebinding of the template to these cavities to perform an indirect quantification [16]. Modification is characterized by template analyte molecules interacting with the selected functional monomer. This relationship between template and functional monomer is similar to *the hand-in-glove principle* [17]. This principle is the factor that ensures the selectivity of the sensor. Compared to environmentally friendly nanomaterials, which are a popular type of modification, MIP technology provides a much more stable surface. Eco-friendly nanomaterials are mostly derived from waste natural compounds[18]. Their structure and composition can be variable and ambiguous, leading to inconsistent results in the field of application. The formation steps of MIPs are more controlled and faster compared to carbon materials, another highly preferred type of modification, because the production of carbon nanomaterials can often be more expensive and complex, especially for nanotubes or specialized forms such as graphene [19]. At the same time, carbon nanomaterials can lose performance over time, often due to surface degradation or changes in electrochemical properties.

Electropolymerization facilitates the formation of polymer layer with distinct morphologies by controlling voltage scan rates and current density in the presence of monomers and template molecules [20]. The construction of polymers with conductive properties with desired conductivity or porosity has demonstrated extraordinary potential for electrochemical analysis [16, 21]. In their study, Karazan and Roushani developed an MIP sensor for the determination of hippuric acid. They performed 10 cycles of electropolymerization using CV technique in a polymerization solution containing m-dihydroxy benzene and o-aminophenol monomers. In addition, the effect of the CV electropolymerization, which are important parameters, on scanning cycles and scanning speeds, the thickness of the MIP film and electrochemical efficiency were examined [22]. Compared to thermal polymerization methods, electropolymerization is faster and usually takes place at room temperature. It does not require additional heat treatment or lengthy polymerization steps. Chemical polymerization methods usually require organic solvents and toxic initiators, while electropolymerization uses fewer chemicals and can often work with water-based electrolyte solutions. It is more in line with the principles of green chemistry. In photopolymerization, on the other hand, the penetration depth of light is limited, so the polymer layers may be more uneven, which can increase the risk of peeling and mechanical instability [16].

Moreover, the desired thickness of the film layer can be accurately controlled by varying the amount of charge passing through the electrode. A non-imprinted polymer (NIP)–based sensor is developed without a template molecule to demonstrate the analyte specificity of the polymer formed and the selectivity of the method. In addition, the IF calculation was made using CIN impurities in the study. It was proven once again that the polymer surface is drug-specific.

A sensor that integrates the benefits of MIP technology with electrochemical techniques for the selective rapid detection of CIN has been developed. The CIN molecule was directly imprinted on the surface of the GCE using electropolymerization, employing *o–*PD as the functional monomer. With the developed sensor, CIN was analyzed for the first time with a MIP-based electrochemical sensor, and the sensor proved its reliability in tablet dosage form and commercial human serum applications. Furthermore, interference studies were conducted with compounds often present in biological fluids and impurities utilized in pharmaceutical formulation research to assess the sensor's selectivity.

## Experimental section

### Reagents and chemicals

CIN standard compounds, impurities (A, B, E, F), and its tablet dosage form (Cineset®) were obtained from NOBEL Holding A.S. (Düzce-Turkey). Glacial acetic acid (≥ 99%), sodium chloride (≥ 99.0%), potassium chloride (99.0–100.5%), sodium acetate trihydrate (≥ 99.5%), ethanol (≥ 99.5%), methanol (≥ 99.8%), sodium hydroxide (≥ 97.0%, pellets), ascorbic acid, caffeine (anhydrous, 99%), glucose, uric acid (≥ 99%), hydrochloric acid (37%), acetonitrile (≥ 99.9%), acetone (≥ 99.5%), potassium ferricyanide (III) (K_3_Fe(CN)_6_) (99%), potassium hexacyanoferrate (II) trihydrate (K_4_Fe(CN)_6_ 3H_2_O) (98.5–102.0%), o–PD (C_6_H_8_N_2_) (99.5%), and commercial human serum samples (AB, male) were purchased from Sigma-Aldrich.

All electrochemical polymerization procedures were conducted in an acetate buffer solution (pH 5.2, ABS), and all voltammetric measurements were performed in a [Fe(CN)_6_]^3−^/^4−^ solution containing 0.1 M KCl. All solutions were obtained with analytical grade reagents and double distilled water from a Millipore Milli-Q system. Every day, the redox probe solution (5 mM [Fe(CN)_6_]^3−/4−^ was newly produced in 0.1 M KCl.

### Instrumentation and equipment

The AUTOLAB-PGSTAT 204 (The Netherlands) equipped with NOVA 2.1.6 software was utilized for all voltammetric measurements. Cyclic voltammograms were obtained at a scan rate of 100 mV s^−1^ within the voltage range of 0 to 0.8 V. DP voltammograms utilized optimal parameters of a 25-mV modulation amplitude, a 0.05-s modulation time, a 5-mV step potential, a 0.8-V stop potential, a 0.005-V s^−1^ sweep rate, and a 5-s equilibrium time. In EIS voltammograms, the parameters were used as first applied frequency 100,000 Hz, 0.01 Hz last applied frequency, 50 frequency number, and 0.01 amplitude. The standard three-electrode configuration employed for the investigations comprises a GCE (BASi, USA; diameter: 3 mm) as the working electrode, Ag/AgCl (3 M NaCl) as the reference electrode, and a platinum wire as the counter electrode.

A SevenCompact™ pH/Ion S220 model (Mettler Toledo, Switzerland) equipped with a combination glass reference electrode was used to calibrate the pH of solutions.

Morphological characterization of the sensor surface was performed using SEM (ZEISS EVO 40) and atomic force microscope (AFM, Nano Magnetics).

### *Formation of polymeric layers for MIP and NIP electrodes *via* electropolymerization*

Before using the bare GCE, it was polished with powdered alumina, washed with ultrapure water, and dried at room temperature as a preliminary preparation. The GCE was also periodically soaked in a 1:1 ratio of methanol:water. To prepare MIP-modified GCE, a monomer solution was prepared in a supportive electrolyte environment comprising CIN and monomer in a molar ratio of 7:1. The electropolymerization mixture was prepared by vortexing *o–*PD (6.3 mg) as a functional polymer, a 10^−3^ M CIN solution (500 μL) as a template molecule, and ABS 5.2 (4.5 mL) as a buffer solution in an Eppendorf tube. The electrochemical polymerization process was conducted in a mixed solution, employing the CV method throughout a potential range of 0 to 0.8 V for 25 cycles at a scan rate of 100 mV/s (Fig. S1). Accordingly, the o–pD facilitates the formation of a powerful electrostatic and hydrogen bonding between the polymeric coating and the electrode surface. To prepare MIP-modified GCE, a monomer solution was prepared in a supportive electrolyte environment comprising CIN and monomer in a molar ratio of 7:1. The template molecule CIN was removed by immersing the imprinted GCE in an ethanol solution at an ambient temperature for 15 min. The MIP-based electrode was then incubated in varying concentrations of CIN for 15 min to facilitate the rebinding process.

A NIP-based sensor was developed without CIN, according to the same necessary procedures used for the MIP sensor. The sensor uses unprinted polymer (NIP) to verify polymer formation and sensor performance.

### Real sample and CIN impurity preparation

The mean weight of 10 tablets, each containing 90.0 mg of CIN, was measured prior to their full pulverization for analysis. An amount of this powder, equivalent to 1 × 10^−3^ M CIN was weighed exactly and made up to 100 mL with ethanol in a balloon jar. The mixture was stirred in an ultrasonic bath for 30 min. Then, an appropriate amount of the upper clear solution was added to the required volume in the tube with the selected support electrolyte. Voltammograms of the prepared solutions were taken under the same conditions as the standard solutions. To demonstrate the method’s accuracy, recovery tests were conducted after the addition of a known quantity of the pure drug ingredient to the drug product’s (tablet) solution. The quantity of the active ingredient was determined using the calibration curve derived from the regression equation.

The performance of the developed sensor in selectively detecting CIN was evaluated with four impurities of CIN, namely, impurity A, impurity B, impurity E, and impurity F. All of the impurity solutions are prepared with a mixture of acetonitrile/water (1:1, v/v).

Commercial human serum samples stored frozen in a refrigerator at − 20 °C were thawed at room temperature. The 10^−3^-M standard serum solution was prepared by adding 1 mL of CIN to 3.6 mL of serum and 5.4 mL of acetonitrile to precipitate the proteins. The sample was then subjected to an ultrasonic bath for 15 min and centrifuged at 5000 rpm for 20 min. The supernatant obtained before the analysis was diluted to the required concentrations with ABS 5.2. To obtain a linear calibration line, a specific concentration range was determined. The serum recovery study was performed with three replicate measurements using the concentration at the midpoint of the calibration line.

## Results and discussion

### Surface characteristics of the MIP sensor

**Fig. 1:**
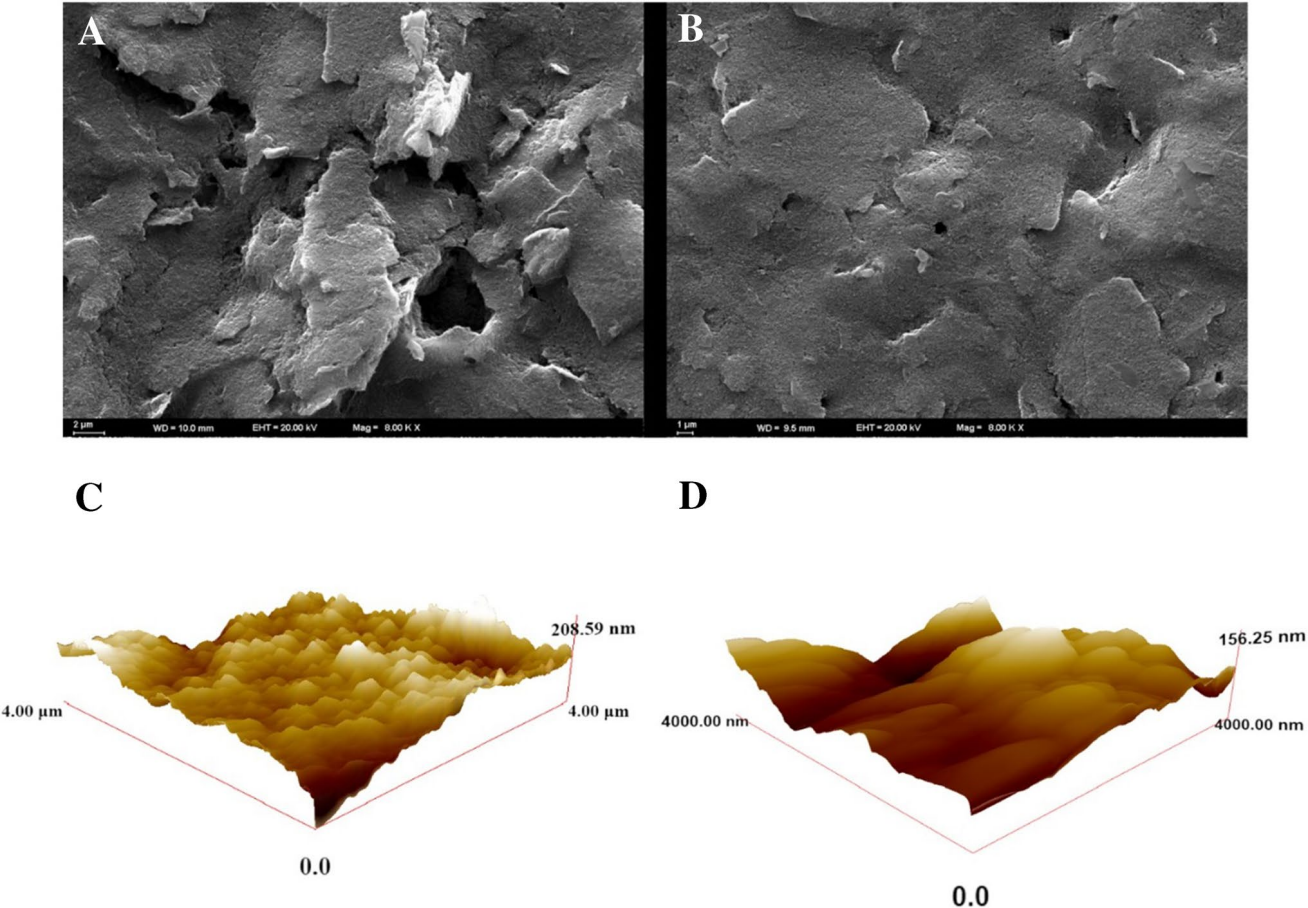
SEM image of CIN@MIP/GCE (**A**) after 15-min ethanol removal and CIN@NIP/GCE (**B**). AFM micrographs of CIN@MIP/GCE(C) after 15-min removal and CIN@NIP/GCE(D)

The surface morphology of the polymeric film on the GCE surface was investigated by SEM. As illustrated in Fig. 1A, the SEM image of the CIN@MIP/GCE sensor displays a porous and rough surface. The morphological characteristics of the CIN@NIP/GCE surface depicted in Fig. 1B display a smoother topography. Following the removal step on the MIP surface, specific cavities are formed on the polymer film surface to facilitate the binding of the template molecule. That is the main contributor to the noticeable disparity between the morphological features of the MIP and NIP surfaces, which in turn confirms the formation of an effective MIP surface. The porous characteristics of the MIP surface enhance the sensor’s selectivity for the rebinding of the template molecule CIN.

In addition, the surface topography of the MIP and NIP sensors was also investigated by the AFM method. Three-dimensional images were recorded in a 4.0 × 4.0-µM^2^ scanning area. Figure 1 C and D show the AFM images of the CIN@MIP/GCE and CIN@NIP/GCE sensors, respectively. According to these images, the surface of the CIN@MIP/GCE sensor, which was prepared by washing with the removal solution shown in Fig. 1C and has CIN-specific voids on its surface, is quite rough. Therefore, the RMS value was found to be 27.95. According to Fig. 1D, a smoother surface formation is observed due to the absence of CIN-specific voids in the CIN@NIP/GCE sensor. This situation is also supported by the RMS value of 21.30 nm.

### Electrochemical characterization of the MIP sensor

**Fig. 2:**
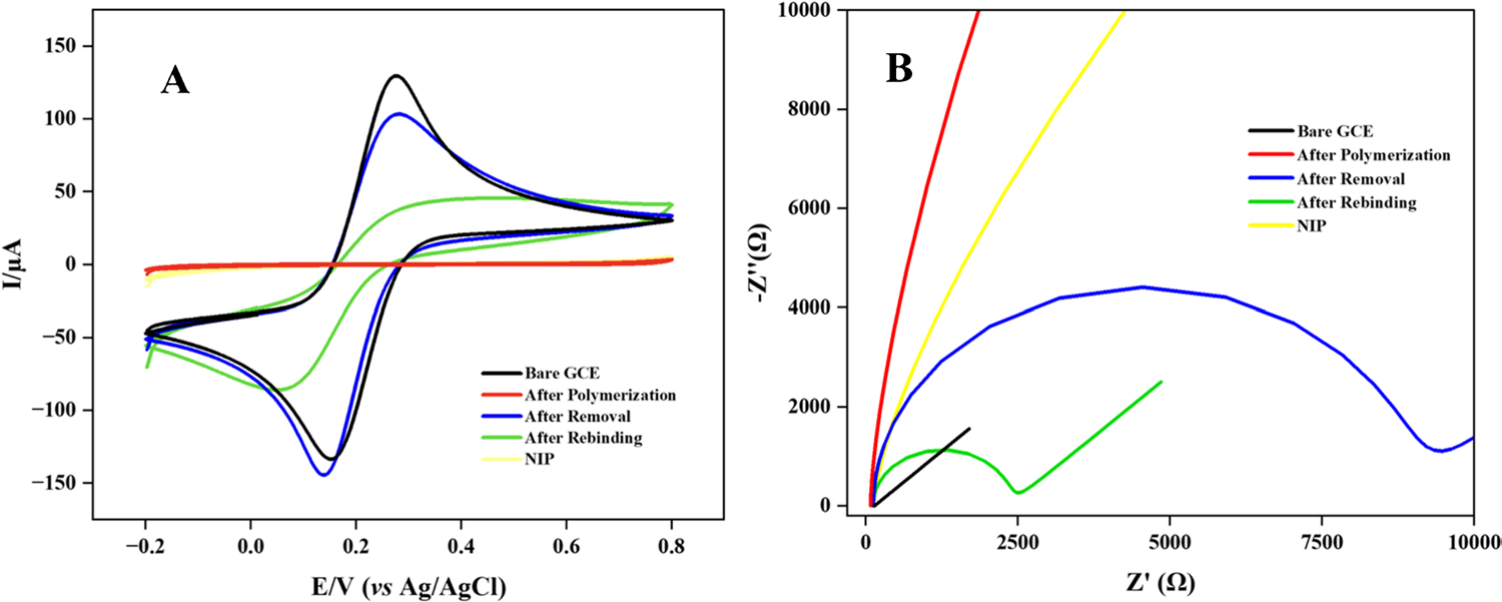
Electrochemical characterization of the bare GCE, after polymerization with *o–*PD, after removal and after rebinding with CIN utilizing CV (**A**) and EIS (**B**)

The electron transport and charge transfer resistance on the CIN@MIP/GCE surface were examined using CV and EIS using a 5-mM Fe(CN)_6_^3−/4−^ redox solution. The electrochemical behavior of the constructed sensor was investigated at several preparation stages, including the bare electrode, after polymerization, after removal, and after rebinding of CIN (Fig. 2). The CV is a crucial method that conveys information regarding distinctive features, including changes in electron transfer rate and sensor conductivity at various steps of preparation [23].

Figure 2 A presents the CV responses of bare GCE (152.3 µA) following polymerization (AP), after removal (AR) (115.2 µA), and rebinding (ARB) (79 µA). The bare electrode exhibited the highest oxidation peak due to the absence of surface modifications that might inhibit electron transfer. The redox probe’s current peak could not be achieved after AP because of the inhibition of the electron transfer by the polymer film on the surface. Upon the removal of CIN from the polymer film, the current peak of the redox probe reemerged due to the formation of cavities on the surface, which facilitated the rebinding of CIN and enabled electron transport. Finally, it was observed that the current peak of the Fe(CN)_6_^3−/4−^ redox solution was significantly reduced when CIN was bound to specific cavities.

EIS is progressively employed in the characterization of sensors and their effective construction by comparing modified and unmodified electrodes [24]. The alterations in charge transfer resistance (*R*_*ct*_) values were interpreted through the results obtained by utilizing Nyquist curves. As seen in Fig. 2B, the lowest *R*_*ct*_ value (81 Ω) was obtained in bare GCE. The polymer film coating on the GCE surface resulted in the formation of an insulating layer that impeded electron transport, thereby elevating the *R*_*ct*_ value (703 kΩ). Upon the removal of CIN, the insulating effect of the polymer film reduced due to enhanced electron transport, resulting in a lower *R*_*ct*_ value (2.56 kΩ). The binding of CIN to specific cavities formed AR-restricted electron transport, resulting in an observed rise in the *R*_*ct*_ value (11.0 kΩ). This finding suggests that CIN selectively binds to the cavities formed on the MIP surface.

In order to explain the rate of redox reactions on the electrode surface, the exchange current density (*I*_0_) values were calculated using the Butler–Volmer equation [25].

1 $${I}_{0}=RT/nF{R}_{ct}$$

In this equation, *R* is universal gas constant (8.314 J mol^−1^ K^−1^), *T* is absolute temperature (K), *n* is the number of electrons involved in the redox process, *F* is Faraday constant (96,485 C mol^−1^), and *R*_*ct*_ is charge transfer resistance (Ω). The *I*_0_ value was calculated as 0.2904 A cm^−2^ for bare electrode, 33 µA cm^−2^ for after polymerization, 0.0098 A cm^−2^ for after removal, and 0.002138 A cm^−2^ for after rebinding. The very low *I*_0_ value after polymerization indicates that the surface is closed and electron transfer is slowed down. The *I*_0_ value increased after removal, indicating an increase in the electrocatalytic activity of the electrode surface and accelerated redox reactions. At the same time, surface capacitance and Warburg impedance values affecting the ionic transport and mass transfer processes occurring at the electrode–electrolyte interface were obtained using electrochemical impedance spectroscopy. All parameters are presented comparatively in Table S1.

In addition, bare GCE, after removal CIN@MIP/GCE, and after rebinding CIN@MIP/GCE surface areas were calculated according to the Randles–Ševčík equation using CV at different scan rates [26, 27].

2 $${I}_{p}=2.686 \times {10}^{5}{n}^{3/2}A c {D}^{1/2}{\upnu }^{1/2}$$

In this equation, *I*_*p*_ is the current of peak, A is electroactive surface area (cm^2^), *D* is diffusion constant (cm^2^/s), *c* is the concentration of the redox solution (mol/cm^3^), *ν* is scan speed (V/s), and *n* is the number that transferred electrons are expressed with [28, 29]. At a scan rate of 100 mV/s, the electrochemical surface area of bare GCE is 0.125 cm^2^, the electrochemical surface area of CIN@MIP/GCE after removal is 0.088 cm^2^, and the electrochemical surface area of CIN@MIP/GCE after rebinding is 0.073 cm^2^. Electroactive surface areas of GCE calculated using CV method at different scan rates are presented in Table S2.

### Optimization parameters for the MIP sensor

A multitude of factors are conducive to the effective construction of a MIP sensor. Optimizing these factors is of great importance for the development of an effective, stable, and selective sensor. The optimized parameters were assessed by analyzing the peak current values of a 5-mM Fe(CN)_6_^3−/4−^ redox solution obtained by utilizing DPV. For this purpose, the current difference (Δ*I*) between the peak current obtained in 5 mM Fe(CN)_6_^3−/4−^ after removal of CIN from the polymer film surface and the peak current obtained after rebinding to the surface was evaluated.

#### The significance of the monomer/template ratio

The ratio of monomer to template is a critical factor influencing the stability and efficacy of the polymer film formed on the GCE surface [30]. In this regard, the effects of 3, 4, 5, 7, and 10 mM concentrations of the *o–*PD functional monomer were investigated. According to Fig. S2 A, when the difference between the DPV signals obtained after AR and ARB of the sensors prepared with functional monomers at different concentrations is compared, it is seen that the highest Δ*I* value was reached with the sensor polymerized with 7 mM *o–*PD. This procedure demonstrated the formation of an effective and stable polymer film, as well as facilitated the removal and rebinding of 10^−4^ M CIN. Therefore, the optimum monomer concentration was selected as 7 mM.

#### Number of electropolymerization cycles for functional monomer

The number of cycles in the polymerization of *o–*PD contributes to the thickness of the polymer film, thereby affecting its stability and processes such as the removal and rebinding of CIN [31]. An excessively thick polymer film may present challenges during removal and rebinding processes due to the potential entrenchment of the CIN within the polymer film. Conversely, a polymer film with insufficient thickness may exhibit inadequate stability. As illustrated in Fig. S2B, the impacts of varying CV numbers (10, 15, 20, 25, and 30 cycles) were evaluated. The Δ*I* value increased up to 25 cycles and then declined as the number of cycles increased. This condition indicated that at cycle numbers beyond 25, the thickness of the polymer layer hindered the removal and rebinding of CIN. This finding prompted the use of 25 cycles in further studies.

#### Removal process of the template molecule

The template molecule’s removal from the polymer film without destroying the surface is contingent upon the selection of an appropriate removal solution and the determination of an appropriate removal time. The effects of different removal solutions and times on the Δ*I* value are shown in Figs. S2 C and D. Optimization studies were carried out with ethanol, acetone, methanol, acetonitrile, and 15 M acetic acid solutions. According to the results obtained, ethanol solution was selected as the removal solution that is effective in the removal of CIN from the surface without damaging the polymer film. After optimizing the removal solution, CIN@MIP/GCE was immersed and kept in the ethanol solution for 5, 10, 15, 20, and 25 min. As the time period was extended, the peak current value correspondingly escalated. After 15 min, a decrease in the peak current was observed due to changes in the homogeneity of the polymer film and the distribution of the cavities. Therefore, the optimum removal time was selected as 15 min.

#### Rebinding process of CIN

The rebinding of the template molecule significantly influences the analytical efficacy and detection time of the sensor. Therefore, the rebinding time is optimized. According to Fig. S2E, CIN@MIP/GCE was kept in 10^−4^ M CIN solution for 10, 15, 20, 25, and 30 min rebinding times. The highest Δ*I* value was obtained in 15 min. After 15 min, a decrease was observed, and the peak current value remained constant. Although CIN was unable to bind to the CIN@MIP/GCE surface cavities with a short rebinding time, longer periods resulted in reduced analytical performance, suggesting that the sensor may have reached saturation. This suggests that 15 min established optimal circumstances for CIN to rebind to the sensor surface.

#### Electroanalytical performance of CIN@MIP/GCE

**Fig. 3:**
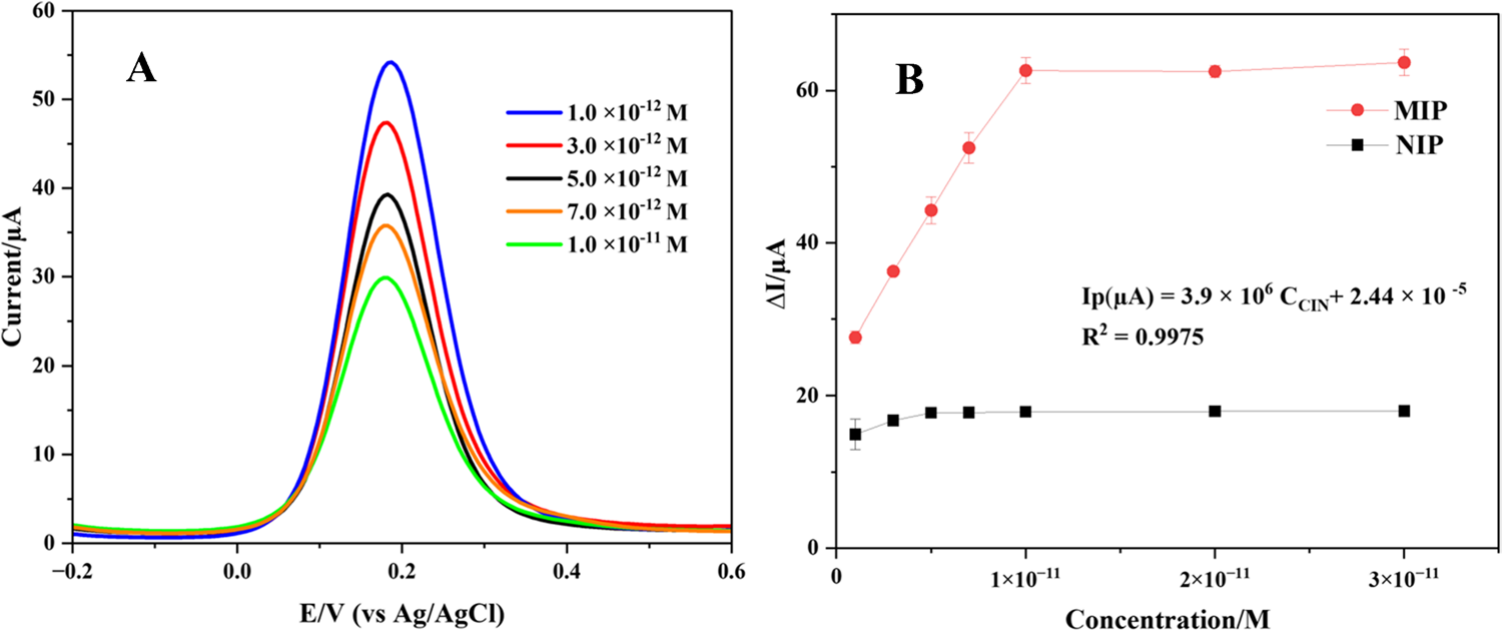
DP voltammograms in 5 mM Fe(CN)_6_^3−/4−^ obtained after rebinding of the different concentrations of CIN in the range of 1.0 × 10^−11^ M to 1.0 × 10^−12^ M to CIN@MIP/GCE (**A**), and the plot of Δ*I* versus the different CIN concentrations obtained by CIN@MIP/GCE and CIN@NIP/GCE (**B**)

The performance of the CIN@MIP/GCE sensor was assessed in a 5-mM Fe(CN)_6_^3−/4−^ redox solution utilizing the DPV approach by preparing the sensor with the required conditions and incubation at different concentrations of CIN (Fig. 3A). The developed sensor was incubated in standard CIN solution at different concentrations. A linearity was obtained for the detection of CIN in the range of 1.0 × 10^−12^ M to 1.0 × 10^−11^ M. In Fig. 3B, the different concentrations of CIN are plotted against Δ*I*. In addition, the CIN@NIP/GCE sensor was prepared as a control electrode to assess the analytical performance and selectivity of the sensor. Its response at different CIN concentrations is given in Fig. 3B. It was noted that an increase in CIN concentration was associated with a linear increase in the Δ*I* value for the CIN@MIP/GCE sensor. Nevertheless, with CIN@NIP/GCE, the Δ*I* value remained relatively constant as the concentration increased. The CIN@MIP/GCE sensor demonstrates efficacy and selectivity in the detection of CIN.

A regression coefficient of 0.9975 was obtained using the equation Δ*I* (µA) = 3.9 × 10^6^ *C* (M) + 2.44 × 10^−5^ for the determination of CIN with CIN@MIP/GCE. Table 1 represents the regression data for the constructed sensor. The LOD was determined to be 0.17 × 10^−12^ M using the equation LOD = 3 s/m, whereas the limit of quantification (LOQ) was obtained as 0.56 × 10^−12^ M using the formula LOQ = 10 s/m [32].

**Table 1:** Regression data of the calibration line for CIN@MIP/GCE

	Standard solution	Serum sample
Linearity range (M)	1.0 × 10^−12^–1.0 × 10^−11^	1.0 × 10^−11^–1.0 × 10^–10^
Slope (μA/M)	3.90 × 10^6^	2.66 × 10^5^
SE slope	1.2 × 10^5^	0.83 × 10^4^
Intercept (μA)	0.244	0.28
SE of intercept	6.77 × 10^−7^	4.36 × 10^–7^
Correlation coefficient (*r*)	0.9978	0.997
LOD (M)	0.17 × 10^−12^	0.9 × 10^−12^
LOQ (M)	0.56 × 10^−12^	0.3 × 10^−11^
Intra-day of peak current repeatability (RSD%)*	0.44	0.32
Inter-day of peak current repeatability (RSD%)*	1.25	1.63

^*^Each value is the mean of three experiments

### Feasibility of CIN@MIP/GCE sensor to real samples

Real sample analyses are crucial for assessing the feasibility of the constructed sensor. The proposed sensor was applied to commercial human serum and tablet dosage forms. The analytical performance of the CIN@MIP/GCE sensor was assessed by adding a predetermined concentration of CIN solution to a predetermined concentration of tablet dosage form solution by the spiking method. The recovery study was conducted to ascertain whether CIN interacted with any of the tablet’s compounds. The recovery values are provided in Table 2. The analysis revealed minimal interference with various components in the pharmaceutical dosage form. The established method demonstrated good accuracy and precision.

**Table 2:** Results of the recovery experiments for serum and tablet samples

	Serum sample	Tablet sample
Label amount (mg)	-	90
Found amount (mg)	-	89.78
RSD%*	-	0.85
Bias%	-	0.24
Spiked amount (mg)	5 × 10^−11^	1.50
Found amount (mg)	5.01 × 10^−11^	1.53
Average recovery (%)	100.19	101.82
RSD %of recovery*	1.12	1.44
Bias%	− 0.19	− 1.82

^*^Each value is the mean of three experiments

**Fig. 4:**
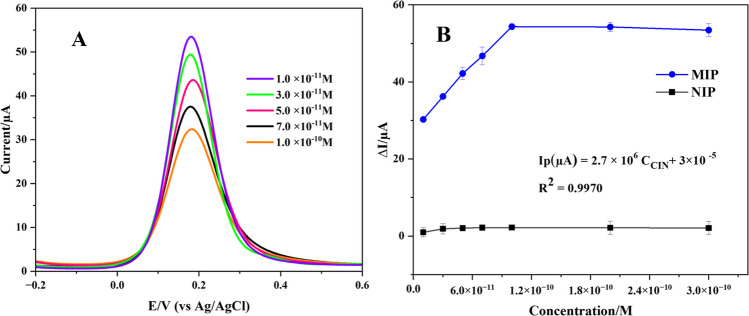
DP voltammograms (**A**), and the plot of ΔI versus the different concentrations of CIN (**B**) for CIN@MIP/GCE in commercial human serum

The CIN@MIP/GCE sensor was applied to diluted commercial human serum samples spiked with CIN. CIN was rebinded to the specific cavities formed on the polymer film surface in the concentration range of 1.0 × 10^−11^–1.0 × 10^−12^ M. Voltammograms obtained with DPV in 5 mM Fe(CN)_6_^3−/4−^ after rebinding CIN of five different concentrations are given in Fig. 4A. A calibration equation as Δ*I* (µA) = 266063 × *C*_CIN_ + 3 × 10^−5^ (*r* = 0.9970) was derived by plotting various CIN concentrations versus Δ*I* within this concentration range (Fig. 4B). Investigations in serum sample were conducted utilizing the CIN@NIP/GCE sensor as a control electrode to assess the analytical accuracy and selectivity of the CIN@MIP/GCE sensor. A linear correlation between CIN concentration and Δ*I* was noted for the MIP sensor, while the NIP sensor demonstrated negligible fluctuation in Δ*I* with changes in concentration. Table 1 summarizes the regression results for the determination of CIN in commercial human serum. The results indicated that the LOD value was 0.9 × 10^−12^ M and the LOQ value was 0.3 × 10^−11^ M. The feasibility of the sensor in the serum sample was also tested by recovery studies (Table 2). The findings substantiated the sensor’s viability, exhibiting remarkable accuracy, precision, and reproducibility.

### Selectivity of CIN@MIP/GCE

Selectivity studies play a crucial role in the validation of the constructed sensor. Selectivity is indicative of the sensor’s efficacy towards the target molecule, despite the presence of structurally analogous compounds. The selectivity features indicate that the target molecule precisely binds with the recognition sites established on the MIP sensor surface. CIN@MIP/GCE exhibits notable selectivity, attributable to the formation of specific binding cavities on the surface that are compatible with CIN. The IF was computed to assess the selectivity of the proposed sensor. For this purpose, the IF calculation was done using the following equation:

3 $$IF=\frac{{\Delta I}_{MIP}}{{\Delta I}_{NIP}}$$

**Fig. 5:**
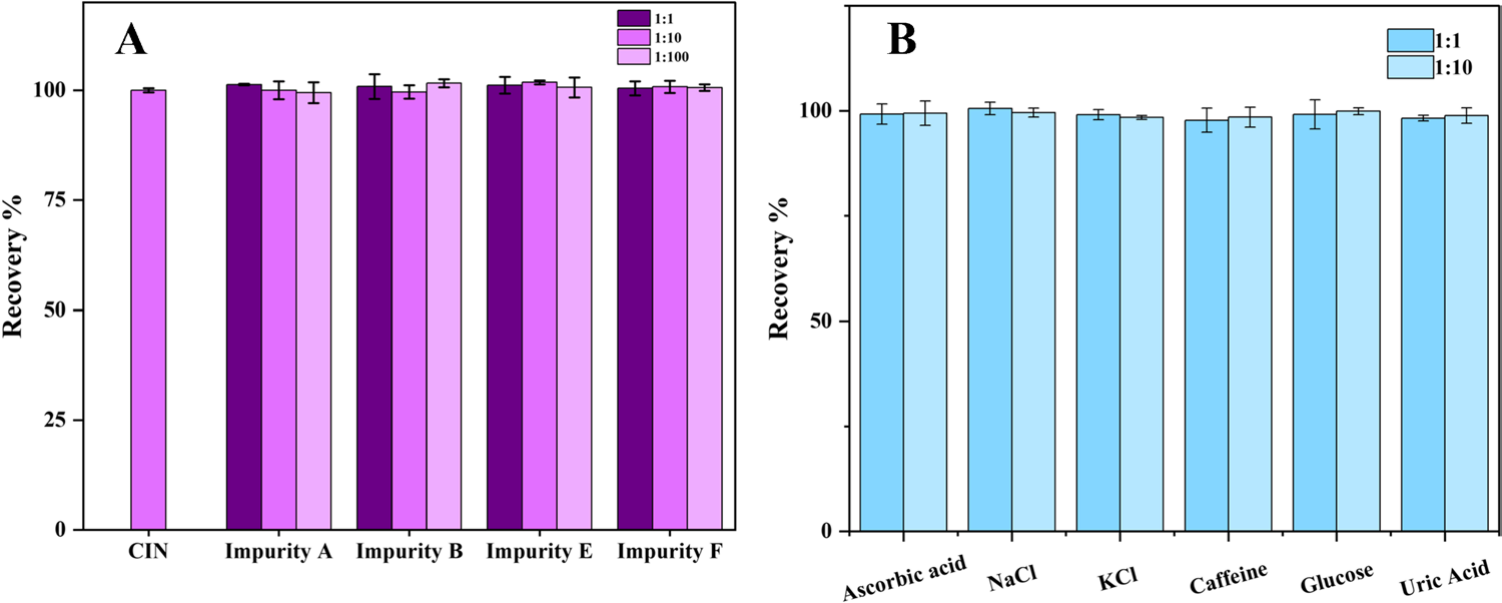
(**A**) Recovery of 5 × 10^−12^ M CIN with 1:1, 1:10, and 1:100 CIN impurities. (**B**) 5 × 10^−12^ M CIN recovery with 1:1 and 1:10 interference compounds

In the equation, Δ*I* is the difference between the current values obtained after the removal and rebinding of CIN, and the Δ*I* values obtained for the MIP and NIP sensors are compared to each other. The selectivity study assessed the efficacy of the CIN@MIP/GCE sensor in solutions with 1, 10, and 100-fold concentrations of impurities A, B, E, and F, along with 5 × 10^−12^ M CIN (Fig. 5A). The chemical structure of CIN and the impurities were given in Fig. S3. IF values obtained as a result of selectivity studies performed with impurities are presented in Table 3. The sensor demonstrated high selectivity for CIN, as indicated by the high IF values for various impurities at different concentrations [33].

**Table 3:** Specificity of MIP/GCE for CIN determination for impurity concentrations of 5.0 × 10^−12^ M, 5.0 × 10^–11^ M, and 5.0 × 10^−10^ M

	Imprinting factor			
Concentration/M	IMP A	IMP B	IMP E	IMP F
5.0 × 10^−12^	5.56	5.62	5.34	5.41
5.0 × 10^−11^	5.24	6.4	5.28	5.97
5.0 × 10^−10^	5.6	6.36	5.63	6.33

### Studies on interference

Interference studies were conducted to assess the selectivity of the sensor. The sensor was observed to be applicable with high selectivity in biological samples in the presence of substances such as ascorbic acid, NaCl, KCl, caffeine, glucose, and uric acid, which are likely to be found in biological fluids. Interference studies were carried out by incubating the CIN@MIP/GCE sensor in solutions prepared by mixing 5 × 10^−12^ M CIN with interfering substances at 1:1 and 1:10 ratios. In comparison with the case of CIN alone, the interference investigations shown in Fig. 5B demonstrate that even at a concentration 10 times higher, the interfering compounds do not significantly alter the peak current responses.

### Sensor stability

CIN@MIP/GCE was preserved to examine the stability of the constructed sensor during a period of 7 days. The improved electrode surface exhibited a stability of 94.4% at the conclusion of the seventh day (Fig. S4). The CIN@MIP/GCE sensor demonstrated good stability after 1 week, keeping about 95% of its reference measurement.

### Comparison with literature

A review of the scientific literature regarding the determination of CIN revealed numerous studies employing chromatographic and spectrophotometric methods for its effective determination. An examination of studies in which CIN determination was performed is provided in Table S1. This table also includes linear concentration ranges, LOD values, and real sample applications. While the determination of CIN was effectively executed in the reviewed studies, the employed methods exhibit certain drawbacks, including high costs, the necessity of preliminary preparation, and prolonged analysis durations [34]. Electrochemical methods offer several benefits, including rapid and cost-effective analytical options, facile sample preparation, and broad applicability [35]. On the other hand, electrochemical approaches demonstrate limited selectivity. MIPs offer a highly selective approach for the analyte by forming cavities that are specific to the target molecule. Accordingly, a highly selective and sensitive electrochemical sensor for CIN determination based on MIP was established in this study.

In comparison to the studies presented in Table S3, the developed CIN@MIP/GCE sensor facilitated the detection of CIN at low detection limits and was effectively utilized for analyzing tablet dosage forms and commercial human serum, yielding high recovery rates. A thorough assessment of the findings reveals that the sensor offers a multitude of advantages, including its practicality, rapid response, environmental sustainability, reliability, high sensitivity, and selectivity. These attributes make it a suitable approach for application in real samples.

## Conclusions

The new MIP-based electrochemical sensor for the sensitive and selective detection of CIN was fabricated using o–PD electrochemical polymerization in the presence of CIN on a GCE. The surface characterization, electrochemical parameters, and analytical effectiveness of the designed CIN@MIP/GCE sensor were evaluated. The sensor demonstrated *linearity* within the range of 1.0 × 10^−12^ to 1.0 × 10^−11^ M, achieving a *sensitivity* with a LOD of 0.17 pg/mL and a LOQ of 0.56 pg/mL. The NIP-based sensor and impurity analyses demonstrated the *selectivity* of the MIP-based sensor designed for CIN. The electrochemical properties of the developed sensor were verified by CV and EIS techniques using a 5-mM [Fe(CN)_6_]^3−/4−^ solution. In addition, surface characterization was examined by SEM and AFM methods. Special cavities created on the polymer film surface to facilitate the binding of the template molecule on the MIP surface were visualized. The sensor’s applicability and accuracy were highlighted by the recovery outcomes and %Bias values derived from its application to pharmaceutical dosage forms and commercial human serum. Intraday and interday repeatability and *precision* of the sensor are demonstrated by %RSD values. In conclusion, the MIP/GCE sensor is distinguished in the literature because of its significant advantages, including rapid analysis time for CIN, ease of application, cost-effectiveness, and superior selectivity.

## Supplementary Information

Below is the link to the electronic supplementary material.

Supplementary file1 (DOCX 1.31 MB)

## Data Availability

No datasets were generated or analysed during the current study.
