# Supplementary material for: A novel electrochemical sensor based on MIP technology for sensitive determination of cinacalcet hydrochloride in tablet dosage form and serum samples
Source: Mikrochim Acta. 2025 Apr 15;192(5):299. doi: 10.1007/s00604-025-07152-7 (PMC12000225; doi:10.1007/s00604-025-07152-7)
Supplement: Supplementary file 1 — Supplementary file1 (DOCX 1.31 MB) [file 604_2025_7152_MOESM1_ESM.docx]

**A Novel Electrochemical Sensor Based on MIP Technology for Sensitive Determination of Cinacalcet in Tablet Dosage Form and Serum Samples**

Ipek Kucuk^a,b^, Selenay Sadak^b,d^, Selda Zengin Kurnalı^c^, Sacide Altınöz^a^, Bengi Uslu^d*^

^a^ Başkent University, Faculty of Pharmacy, Department of Analytical Chemistry,06790, Ankara, Türkiye

^b^ Ankara University, The Graduate School of Health Sciences, 06110, Ankara, Türkiye

^c^ NOBEL Holding A.S. R&D Center, 81100,Düzce, Türkiye

^d^ Ankara University, Faculty of Pharmacy, Department of Analytical Chemistry, 06560, Ankara, Türkiye

**Abstract**

Cinacalcet hydrochloride (CIN) is a calcium-sensing receptor agonist used to treat hypercalcemia in the parathyroid. The molecularly imprinted polymer (MIP) based sensor (CIN@MIP/GCE) was electropolymerized using cyclic voltammetry (CV) of the functional monomer o-phenylenediamine (o-PD) with a template molecule CIN on a glassy carbon electrode (GCE). The optimum performance of the MIP-based electrode for CIN detection was obtained with parameters of a 1:7 monomer ratio, a 15 min removal time, ethanol as a removal solution, and a 15 min rebinding time. The surface characterization of the CIN@MIP/GCE sensor was conducted using atomic force microscopy (AFM) and scanning electron microscopy (SEM), while CV and electrochemical impedance spectroscopy (EIS) were employed for electrochemical characterization with [Fe(CN)_6_]^3-^/^4–^ redox probe. AFM findings show that the MIP sensor with CIN-specific voids on the surface has a root-mean-square (RMS) value of 27.95, while the non-imprinted polymer (NIP) sensor without voids has a smoother surface formation and an RMS value of 21.30 nm. The analytical efficacy of the constructed sensor was assessed using differential pulse voltammetry (DPV). The limit of detection (LOD) was calculated as 0.17×10 ^-12^, with a linear range of 1.0 ×10 ^-12^ – 1.0 × 10^-11^ M. The reliability of the constructed sensor was determined using CIN detection in real samples as tablet dosage form and human serum, yielding recovery results of 100.19% and 101.82%, respectively. The selectivity investigation was performed against prevalent interference substances. The relative imprinting factor (IF) values of CIN impurities confirmed the selectivity of the CIN sensor.

*Corresponding author email: [*buslu@pharmacy.ankara.edu.tr*](mailto:buslu@pharmacy.ankara.edu.tr)

***Keywords:*** *Cinacalcet hydrochloride, Imprinting factor, Molecularly imprinted polymers,* *Pharmaceutical dosage form, Sensor,*


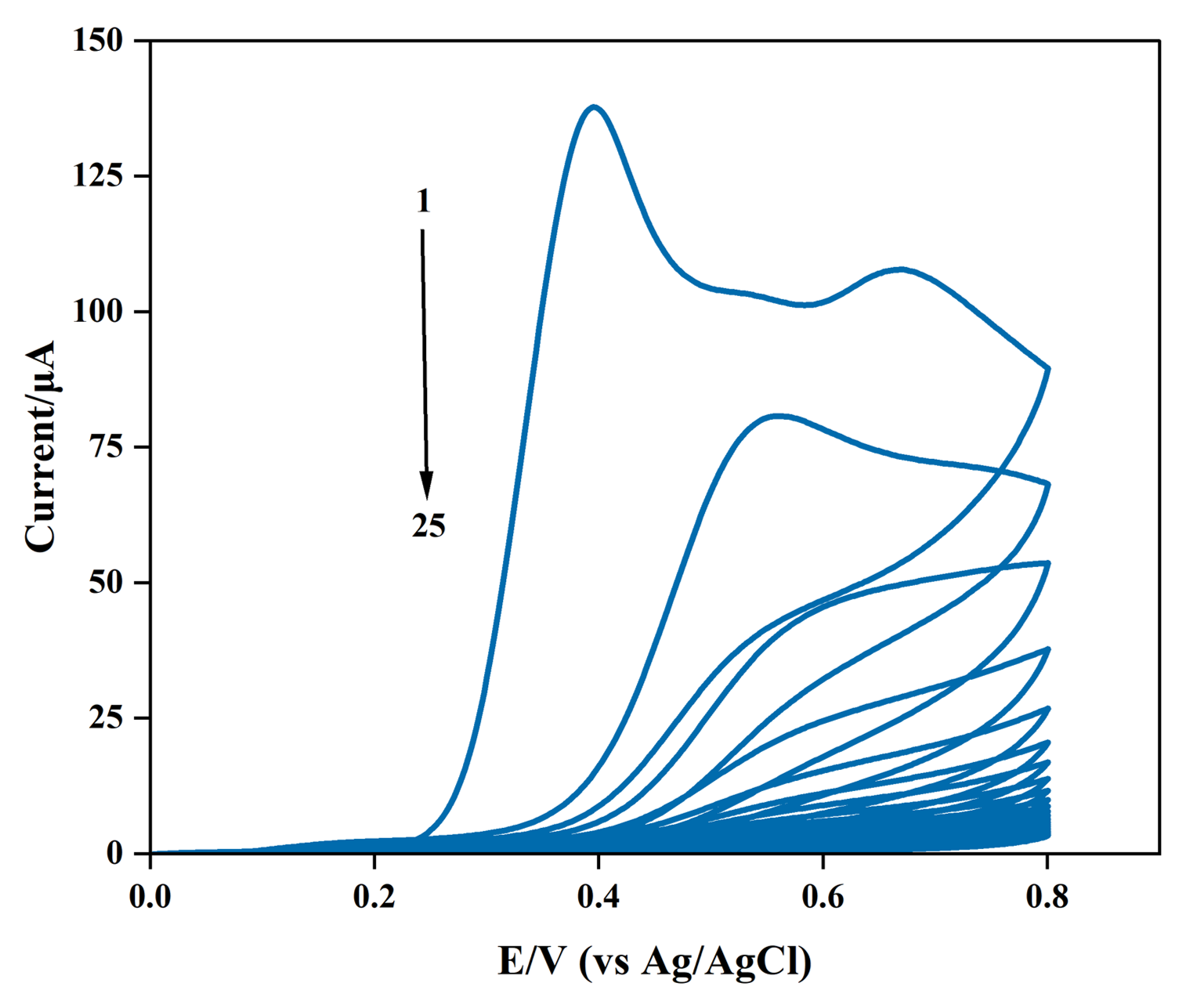


**Fig. S1.** Electrochemical polymerization with 25 cycles utilizing CV on bare GCE.


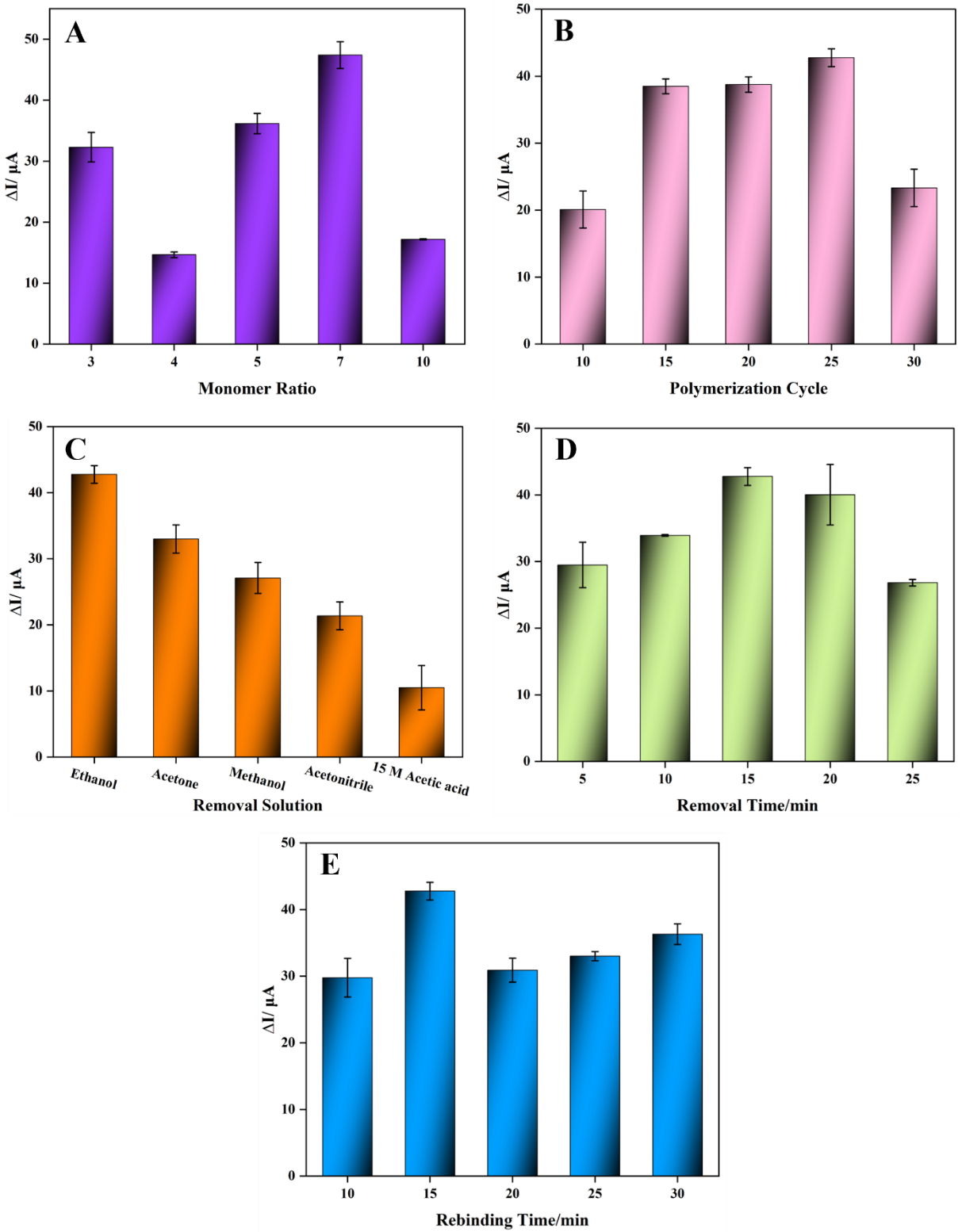


**Fig.S2.** The plot of ΔI values obtained after removal and rebinding of 10^-4^ M CIN at different monomer concentrations (A), number of polymerization cycles (B), removal solutions (C), removal time (D), and rebinding time (E).

**
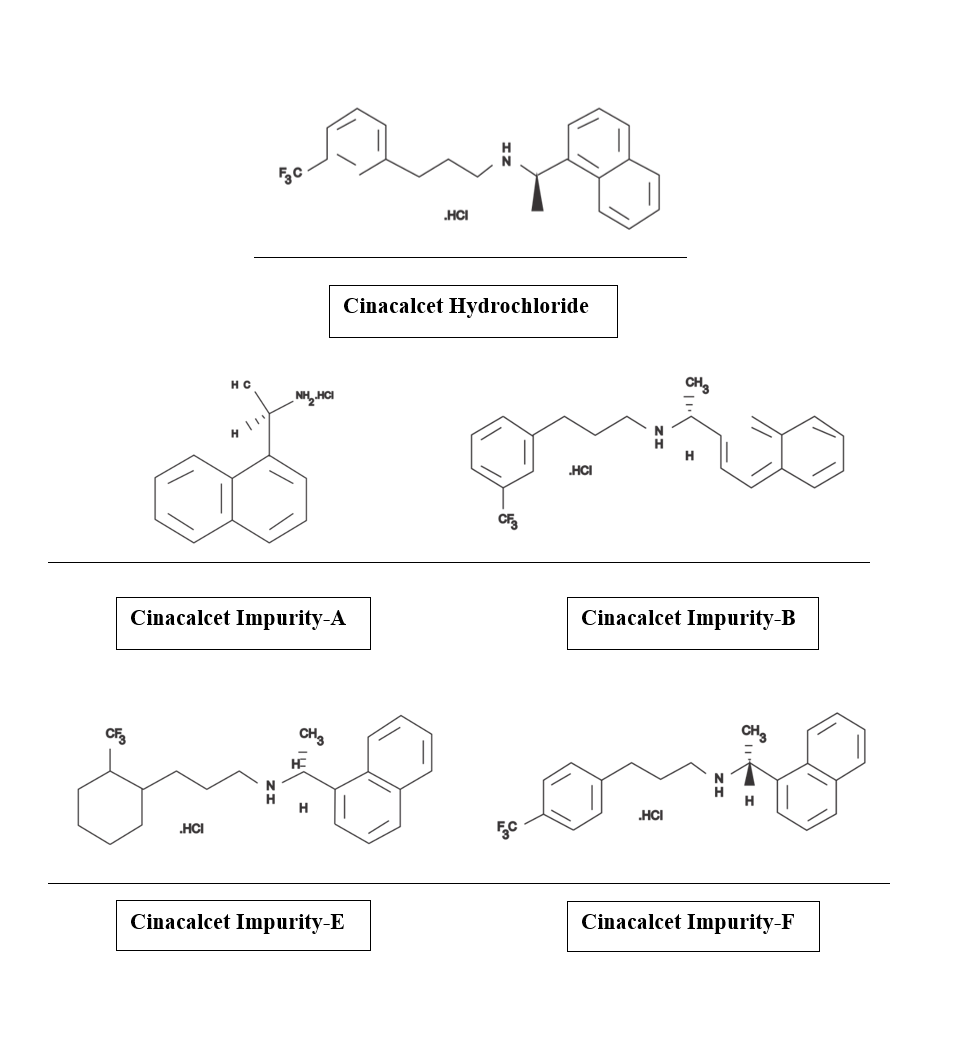
**

**Fig.S3.** The chemical structures of cinacalcet hydrochloride and the impurities belonging to cinacalcet.


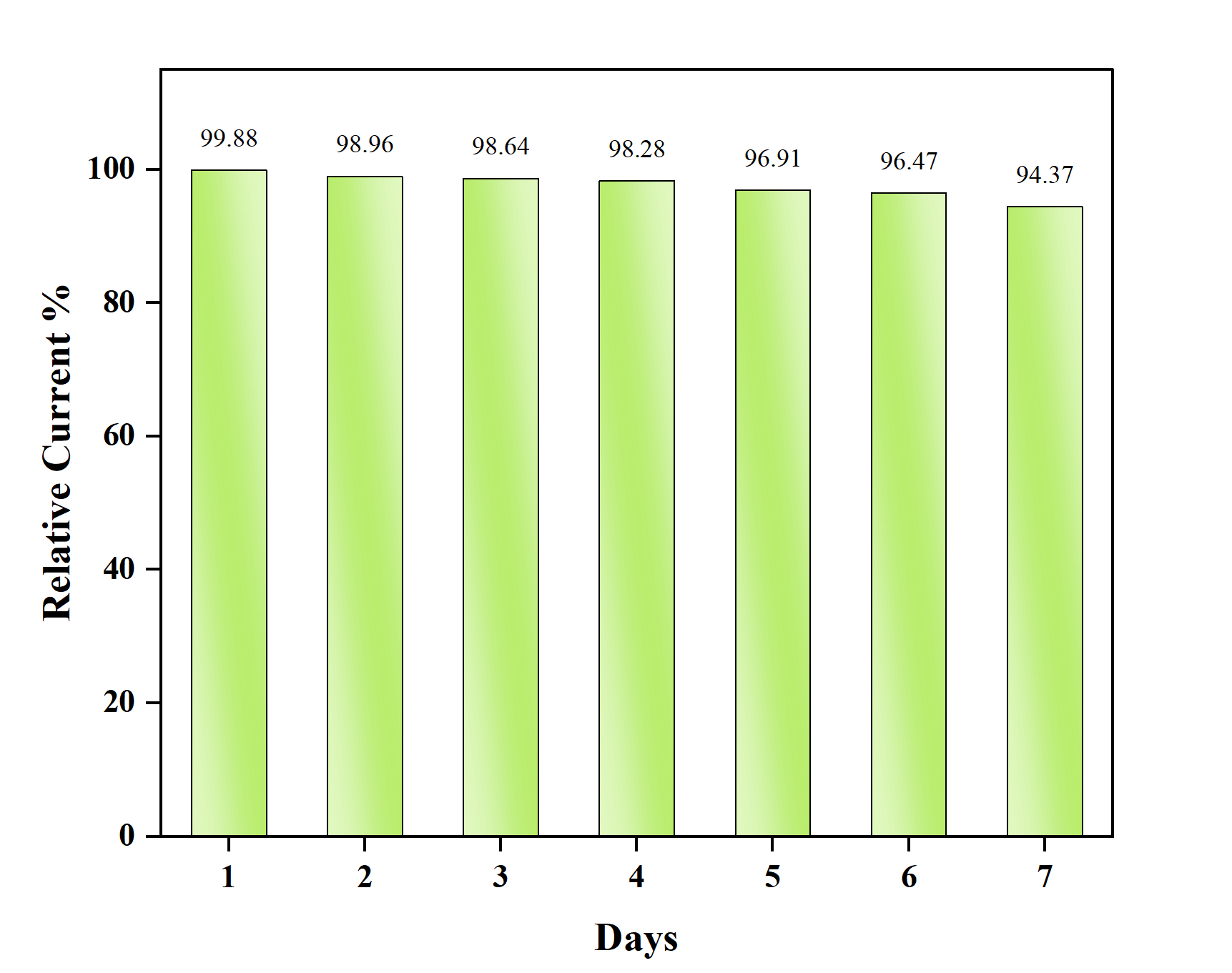


**Fig. S4.** The stability of the CIN@MIP/GCE.

**Table S1.** Comparison of impedance parameters.

|  | Capacitance  (C) | Warburg Impedance  (Zw) | Charge Transfer Resistance  (Rct) | Exchange Current Density (I_0_) |
| --- | --- | --- | --- | --- |
| Bare GCE | 0.04 F | 4.09 µ Ω .s^-1/2^ | 81 Ω | 0.2904 A cm^-2^ |
| After Polimerization | 2.05 µF | 0.001368 Ω .s^-1/2^ | 703 kΩ | 33 µA cm^-2^ |
| After Removal | 6.89 µF | 10.4 µ Ω .s^-1/2^ | 2.56 kΩ | 0.00918 A cm^-2^ |
| After Rebinding | 4.11 µF | 767 µ Ω .s^-1/2^ | 11 kΩ | 0.002138 A cm^-2^ |

**Table S2.** Electroactive surface areas of different scan rates.

| **Scan rate**  **(mV s^-1^)** | **Bare GCE** | **After Removal** | **After Rebinding** |
| --- | --- | --- | --- |
| **10** | 0.12877 | 0.119557 | 0.084378 |
| **25** | 0.129545 | 0.102143 | 0.097099 |
| **50** | 0.127934 | 0.095529 | 0.084302 |
| **75** | 0.126354 | 0.092842 | 0.06961 |
| **100** | 0.124902 | 0.088806 | 0.073439 |
| **150** | 0.122646 | 0.08273 | 0.070488 |
| **200** | 0.120763 | 0.082654 | 0.075123 |
| **250** | 0.119183 | 0.076404 | 0.072949 |

**Table S3.** Comparison with CIN analysis studies in the literature

| Sensing Technique | Linear range | Lod | Application | Recovery (%) | Ref |
| --- | --- | --- | --- | --- | --- |
| LC-MS/MS | 0.1-50 ng/mL | 0.1 ng/mL | Plasma | 85-115 | [2] |
| LC-MS/MS | 0.1-200 ng/mL | 0.1 ng/mL | Plasma | 98.5-103.7 | [3] |
| LC-MS/MS | 0.05-20 ng/mL | 0.05 ng/mL | Plasma | 96-106 | [4] |
| LC-MS/MS | 01.-100 ng/mL | 0.1 ng/mL | Plasma | 90-106 | [5] |
| RP-HPLC | 5-50 µg/mL | 0.32 µg/mL | Tablet | 99.85-100.14 | [6] |
| RP-HPLC | 0.2-5.5 ng/mL | 0.75 ng/mL | Plasma | 101-102 | [7] |
| HPLC-UV | 5-5000 ng/mL | 2.5 ng/mL | Plasma | 95.06-99.47 | [8] |
| HPLC-UV | 1-24 µg/mL | 1 µg/mL | Rat plasma | 82.80-104.08 | [9] |
| UV via derivatization | 3-100 µg/mL | 1.9 µg/mL | Tablet | 100.8-102.23 | [10] |
| RP‑UFLC | 100-100.000 ng/mL | 3193.66 ng/mL | Serum | 98-102 | [11] |
| CIN-MIP/GCE | 0.01-0.001 ng/mL | 0.17 pg/mL | Tablet, and serum | 100.19-101.82 | **This study** |
